# White matter damage and systemic inflammation in Parkinson’s disease

**DOI:** 10.1186/s12868-017-0367-y

**Published:** 2017-06-08

**Authors:** Pi-Ling Chiang, Hsiu-Ling Chen, Cheng-Hsien Lu, Pei-Chin Chen, Meng-Hsiang Chen, I.-Hsiao Yang, Nai-Wen Tsai, Wei-Che Lin

**Affiliations:** 1 Department of Diagnostic Radiology, Kaohsiung Chang Gung Memorial Hospital, Chang Gung University College of Medicine, 123 Ta-Pei Road, Niao-Sung, Kaohsiung, 83305 Taiwan; 2 Department of Neurology, Kaohsiung Chang Gung Memorial Hospital, Chang Gung University College of Medicine, Kaohsiung, Taiwan; 3 Department of Biological Science, National Sun Yat-Sen University, Kaohsiung, Taiwan

**Keywords:** Systemic inflammation, White matter damage, Diffusion tensor imaging, Parkinson’s disease

## Abstract

**Background:**

Systemic inflammation and white matter (WM) alterations have been noted as effects of Parkinson’s disease (PD). This study sought to evaluate WM integrity in PD patients using diffusion tensor imaging (DTI) and to assess its relationship with systemic inflammation.

**Methods:**

Sixty-six patients with PD (23 men and 43 women) and 67 healthy volunteers (29 men and 38 women) underwent blood sampling to quantify inflammatory markers and DTI scans to determine fiber integrity. The inflammatory markers included leukocyte apoptosis, as well as cellular and serum adhesion molecules, in each peripheral blood sample. DTI-related indices [including fractional anisotropy (FA), axial diffusivity (AD), radial diffusivity (RD), and mean diffusivity (MD)] were derived from DTI scans. The resulting FA maps were compared using voxel-based statistics to determine differences between the PD and control groups. The differences in the DTI indices, clinical severity, and inflammatory markers were correlated.

**Results:**

Exploratory group-wise comparison between the two groups revealed that the PD patients exhibited extensive DTI index differences. Low FA accompanied by high RD and MD, without significant differences in AD, suggesting a demyelination process, were found in the parietal, occipital, cerebellar, and insular WM of the PD patients. The declined DTI indices were significantly correlated with increased clinical disease severity, adhesion molecules, and leukocyte apoptosis.

**Conclusions:**

Patients with PD experience WM integrity damage in vulnerable regions, and these impairments are associated with increased disease severity and systemic inflammation. The possible interactions among them may represent variant neuronal injuries and their consequent processes in PD.

**Electronic supplementary material:**

The online version of this article (doi:10.1186/s12868-017-0367-y) contains supplementary material, which is available to authorized users.

## Background

Parkinson’s disease (PD) is a neurodegenerative disease characterized by dopamine (DA) neuron loss in the substantia nigra (SN). It is believed that neuroinflammatory processes contribute to the development of PD [1]. The alterations of white matter (WM) in PD are associated with cognitive impairment, and such alterations may be useful in assessing the onset of dementia in PD patients [2]. Although neuroinflammatory and WM damage play an important role in the development of PD, the relationship between systemic inflammation and WM alterations related to PD has not been systematically investigated with critical insights.

Microglia-associated neuroinflammation in the SN contributes to the development of PD [3] and may be stimulated by systemic inflammation. Several peripheral blood leukocyte adhesion molecules have been reported as contributing to a connection between systemic inflammation and neuroinflammation in PD, including macrophage antigen complex-1 (Mac-1) [4], lymphocyte function-associated antigen 1 (LFA-1) [5], E-selectin [6], and P-selectin [7]. These adhesion molecules also contribute to traffic of the activated T cells across the blood–brain barrier [8]. By evaluating peripheral blood leukocyte apoptosis, Lin et al. and others [9, 10] have demonstrated its possible relationship with central striatum neural loss by using SPECT imaging. In addition, increased inflammation has also been found to be associated with WM damage in different neurological diseases [11]. It is thus reasonable to hypothesize that increased systemic inflammation might also alter the integrity of WM in PD. Furthermore, we wanted to obtain the microglial activation via MAC-1 [12], and to obtain the infiltration of peripheral immune cells via measuring the LFA-1 [13], leukocyte apoptosis [10] and selectin category of adhesion molecular.

Previous clinical studies have used diffusion tensor imaging (DTI) to describe WM integrity alterations in PD [14]. Among various quantitative parameters derived from DTI, fractional anisotropy (FA) values, mean diffusivity (MD) values, radial diffusivity (RD) values, and axial diffusivity (AD) values are recognized as the most useful for evaluating the integrity of WM fibers. Changes in WM integrity in PD identified through the use of DTI have previously been reported, including a widespread pathology of cerebral WM identified through reduced FA values and/or increased MD and RD values [14–16]. However, the correlation, if any, between particular WM alterations and systemic inflammation in PD has still not been well evaluated.

In technology, the voxel-based statistics and the tract-based spatial statistics (TBSS) are both FA data analysis tools to localize brain changes related to development, degeneration and disease. The former is compromised by voxel to voxel in a region of interest or whole brain, and the latter is compromised by the use of standard registration algorithm, the alignment-invariant tract estimated by FA data (the “mean FA skeleton”) [17, 18]. Using TBSS analysis, some white matter abnormalities, such as locally reduce FA, may be not so accurate due to extra-tract voxels. Furthermore, the accuracy relies on the accurate FA skeleton [19]. So we chose the voxel-based statistics in this study.

In the present study, we hypothesized that the increased systemic inflammation observed in patients with PD correlates with the WM damage. The purposes of this study were (1) to evaluate WM integrity in PD patients using DTI; (2) to investigate the differences in systemic inflammation and WM integrity between patients with PD and healthy controls; and (3) to evaluate the relationships between WM alterations and systemic inflammation.

## Methods

### Patients

This prospective study enrolled 66 patients (23 men and 43 women; mean age, 58.1 ± 8.7 years; mean levodopa dosage 278.97 ± 327.07 mg; mean disease duration 3.856 ± 3.588 years) with idiopathic PD diagnosed by an experienced neurology specialist in the Neurology Department of Chang Gung Memorial Hospital according to the United Kingdom Brain Bank criteria [20], and without a history of other neurologic or psychiatric illness or psychotropic medication usage. The PD criteria included (1) bradykinesia and at least 1 of the following symptoms including muscular rigidity, rest tremor and postural instability; (2) exclusion of other causes of parkinsonism; and (3) at least three of the following supportive criteria: unilateral onset, the presence of rest tremor, progressive disorder, persistent asymmetry most affecting the side of onset, excellent response (70–100%) to levodopa, severe levodopa-induced chorea, levodopa response for 5 years or more, and clinical course of 10 years or more. For comparison, 67 sex- and age-matched healthy volunteers (29 men and 38 women; mean age, 56.8 ± 9.8 years) with similar levels of education, and without a medical history of neurologic disease or psychiatric illness, alcohol or substance abuse, or head injury, were recruited as a control group. The Chang Gung Memorial Hospital Ethics Committee approved the study, and all of the participants provided written informed consent.

The disease severity and functional status of each patient were evaluated using the Unified Parkinson’s Disease Rating Scale (UPDRS) [21], the modified Hoehn and Yahr Staging Scale (modified H & Y scale) [22], and the Schwab and England Activities of Daily Living Scale (S & E scale) [23].

### Blood sampling and laboratory investigations

Systemic inflammation was measured in terms of the percentage of apoptotic peripheral leukocytes and the plasma levels of adhesion molecules. Blood was drawn by forearm venipuncture on the same day as the MRI study. The evaluators of clinical and immunological data were blinded to the other data and MRI data.

#### Assessment of leukocyte apoptosis

Detailed description of the assessment of leukocyte apoptosis, cellular adhesion molecules, and serum adhesion molecules were presented in our previous studies [24, 25]. The condition of leukocyte apoptosis was assessed with APO 2.7-phycoerythrin (PE) (clone 2.7A6A3; Immunotech), with the positive expression of APO 2.7-PE indicating the presence of apoptotic cells. A 100 μL whole blood sample was stained with 10 μL CD45-phycoerythrin (PE)-Cy5 (clone J33; Immunotech, Marseille, France) at room temperature for 15 min. The CD45-PE-Cy5 antibody reacted with the CD45 family of transmembrane glycoproteins, a panleukocyte marker expressed on the surface of all human leukocytes. The blood cells were fixed using 5.5% formaldehyde, washed with phosphate buffered saline, and then incubated with IntraPrep permeabilization reagent (Immunotech) for 5 min without shaking, while the cells made contact with the APO 2.7-PE. Mouse immunoglobulin G (IgG)-PE was used as a control for nonspecific staining. All the leukocytes and their subtypes, which were identified based on the intensity of CD45 expression, were analyzed by flow cytometry. A database coordinator monitored all data collection and entry, both of which were checked for any inconsistencies.

#### Assessment of adhesion molecules

The quantities of cellular adhesion molecules were expressed as the mean fluorescence intensity (MFI) of antibody-positive leukocytes. The samples were simultaneously incubated with saturating concentrations of PE-conjugated antibodies against LFA-1 or Mac-1 (Becton–Dickinson Biosciences) and PE-Cy5-labeled antibodies against CD45 (clone VI-PL2). Leukocytes were incubated with PE-coupled unspecific mouse IgG1 (Becton–Dickinson Biosciences) as control experiments. After immuno-labeling, commercial lysing buffer (BD Biosciences Pharmingen) was added to lyse red blood cells, and then paraformaldehyde was added for fixation. Anti-LFA-1 or anti-Mac-1 antibodies were then determined by analyzing 10,000 leukocytes for PE-positive fluorescence. The results were expressed as the MFI of antibody-positive leukocytes.

Serum P-selectin, E-selectin, and L-selectin levels were assessed using commercially available enzyme-linked immunosorbent assays (R&D Systems, Minneapolis, MN, USA) as previously described [26]. In these assays, the samples were incubated in microtitration wells coated with anti-P-selectin, E-selectin, and L-selectin antibodies, after which another antiantigen detection antibody labeled with enzyme horseradish peroxidase was added. After that, the wells were incubated with substrate tetramethylbenzidine, and then acidic stopping solution was added. The concentration of antigens present was directly proportional to the absorbance at 450 and 620 nm determined by a dual-wavelength absorbance measurement.

### MRI data acquisition

#### Data acquisition

For each subject, an MRI scan was performed using a 3.0 Tesla whole-body GE Signa MRI system (General Electric Healthcare, Milwaukee, WI, USA) equipped with an eight-channel head coil. DTI was conducted along the anterior-posterior commissure line in the axial plane using a single shot spin-echo echoplanar imaging sequence (repetition time/echo time = 15,800/77 ms, number of excitations = 3, matrix size = 256 × 256, field of view = 25.6 cm, voxel size = 1 × 1 × 2.5 mm^3^, 55 slices without gaping). The diffusion images gradient encoding schemes included 13 noncollinear directions with a b-value of 1000 s/mm^2^ and a nondiffusion weighted image volume b-value of 0 s/mm^2^.

#### Data preprocessing

A detailed description of DTI data preprocessing was presented in our previous study [24]. The magnetic resonance images were preprocessed using the FSL v5.0.4 [Functional Magnetic Resonance Imaging of the Brain (FMRIB) Software Library, Oxford, UK; http://www.fmrib.ox.au.uk/fsl], including eddy current correction and brain tissue extraction [27]. The details of our preprocessing pipeline have been previously reported [24] and posted online as Additional file 1: Supplementary methods. A diffusion tensor model was fitted in each voxel using FMRIB’s Diffusion Toolbox for the calculation of FA values; AD values, λ1; RD values (λ2 + λ3)/2; and MD values (λ1 + λ2 + λ3)/3.

### Statistical analysis

#### Analysis of demographic data differences between groups

The statistical analyses were performed using the Statistical Package for Social Sciences (SPSS) software package (version 17, SPSS Inc. Chicago, IL, USA). Age data for the study groups was compared using the independent t test. Sex data for the study groups was compared using the Pearson Chi square test. Analysis of covariance (ANCOVA) with age and sex as potential confounding variables was used to compare the group differences in terms of years of education. ANCOVA with age and sex as potential confounding variables was used to compare the group differences in laboratory data in terms of leukocyte apoptosis and adhesion molecules. Statistical significance was set at P < 0.05.

#### Analysis of group comparison of FA values

Statistical analyses were conducted using the SPM8 (Statistical Parametric Mapping; http://www.fil.ion.ucl.ac.uk/spm/; University College London, London, UK) software package, which itself used Matlab R2010a (Mathworks, Natick, MA, USA) for voxel-wise group comparisons. Smoothed, normalized FA images were analyzed using SPM8 within the framework of a general linear model, whereas ANCOVA was performed with age, sex, and years of education as covariates to investigate the FA differences between the PD and control groups. The FA threshold of the mean WM was set at 0.2 to successfully exclude voxels, which consisted of gray matter (GM) or cerebrospinal fluid in most subjects. The resultant statistical inferences were considered significant under the criteria of cluster level family-wise error (FWE) corrected P value <0.05, with a cluster size of at least 610 voxels, based on the results of the Monte Carlo simulation (3dClusterSim with the following parameters: single voxel P value <0.001, FWHM = 7 mm with GM mask, and 10,000 simulations). The most probable fiber tracts and anatomic locations of each significant cluster were determined using the FSL atlas tool (http://www.fmrib.ox.ac.uk/fsl/fslwiki/Atlases).

#### Analysis of region of interest

Region of interest (ROI) analyses were conducted to determine the mean FA value of each significantly different area between the two groups based on whole-brain voxel-wise comparisons. The Marsbar toolbox (http://marsbar.sourceforge.net/download.html) was used to extract the ROI masks. The mean DTI-related indices (including FA, AD, RD, and MD) of these areas were compared between groups by multivariate analysis of covariance, with age, sex, and years of education as covariates. Significance was set at a Bonferroni corrected P < 0.05, accounting for multiple ROI comparisons.

#### Relationship among regional DTI-related indices, clinical severity, and inflammation parameters

Partial correlation analysis adjusted for age, sex, and years of education was performed to correlate the clinical severity and inflammation parameters with the regional DTI-related indices in the patient group. The threshold for statistical significance was set at P < 0.05, with Bonferroni correction for multiple comparisons. Furthermore, linear regression analysis adjusted for age and sex were performed for a more complete modeling of the relationship between systemic inflammation and WM changes.

## Results

### Demographic characteristics and inflammation parameters

The demographic characteristics and systemic inflammatory results of the two groups, and clinical severity results of the PD group are listed in Table 1. There were no significant differences in age, sex, and years of education between the two groups. The leukocyte apoptosis, Mac-1, LFA-1, and serum P-selectin values were significantly higher in the PD group than in the controls (P < 0.05).

**Table 1:** Demographic data and oxidation parameters of patients with PD and controls

	PD (n = 66)	Control (n = 67)	*P*
*Clinical demographics*			
Age (year)	58.1 ± 8.7	56.8 ± 9.8	0.808
Sex (M, F)	23, 43	29, 38	0.319
Years of education	9.45 ± 4.53	11.21 ± 4.87	0.178
UPDRS I	3.08 ± 2.65		
UPDRS II	10.42 ± 10.82		
UPDRS III	22.74 ± 16.76		
UPDRS total	35.15 ± 26.23		
Modified H & Y	1.98 ± 1.11		
S & E	85.15 ± 17.82		
Levodopa dosage (mg)	278.97 ± 327.07		
*Inflammation parameters*			
Granulocyte APO 2.7 (%)	1.13 ± 0.97	0.77 ± 0.90	0.021
Monocyte APO 2.7 (%)	5.56 ± 6.33	2.75 ± 3.37	0.002
Lymphocyte APO 2.7 (%)	0.72 ± 0.49	0.46 ± 0.37	0.001
Total leukocyte APO 2.7 (%)	1.78 ± 1.24	1.14 ± 1.05	0.002
Granulocyte Mac-1	53.71 ± 23.83	42.43 ± 13.91	0.001
Granulocyte LFA-1	6.34 ± 1.31	5.67 ± 1.44	0.002
Monocyte Mac-1	57.38 ± 30.75	42.27 ± 22.48	0.002
Monocyte LFA-1	18.46 ± 5.33	14.69 ± 4.74	0.000
Lymphocyte Mac-1	10.87 ± 3.51	9.31 ± 2.23	0.004
Lymphocyte LFA-1	14.91 ± 3.41	12.78 ± 3.25	0.000
P-selectin	100.03 ± 20.26	86.88 ± 22.00	0.000
L-selectin	887.75 ± 171.67	913.43 ± 226.79	0.656
E-selectin	33.73 ± 15.17	35.71 ± 17.68	0.462

Sex data were compared by Pearson Chi square test. Age data were compared by independent t test. Year of education data were compared by analysis of covariance (ANCOVA) after controlling for age and sex. Inflammation parameters data were compared by ANCOVA after controlling for age and sex

Data are presented as mean ± standard deviation

*UPDRS* Unified Parkinson’s Disease Rating Scale, *Modified H & Y* Modified Hoehn and Yahr stages (maximum score is 5), *S & E* Schwab and England activities of daily living scale (minimum score is 0, suggesting vegetative functions), *APO* apoptosis, *Mac*-*1* macrophage antigen-1, *LFA*-*1* leukocyte function associated antigen-1

### Regional WM integrity aberrances

**Fig. 1:**
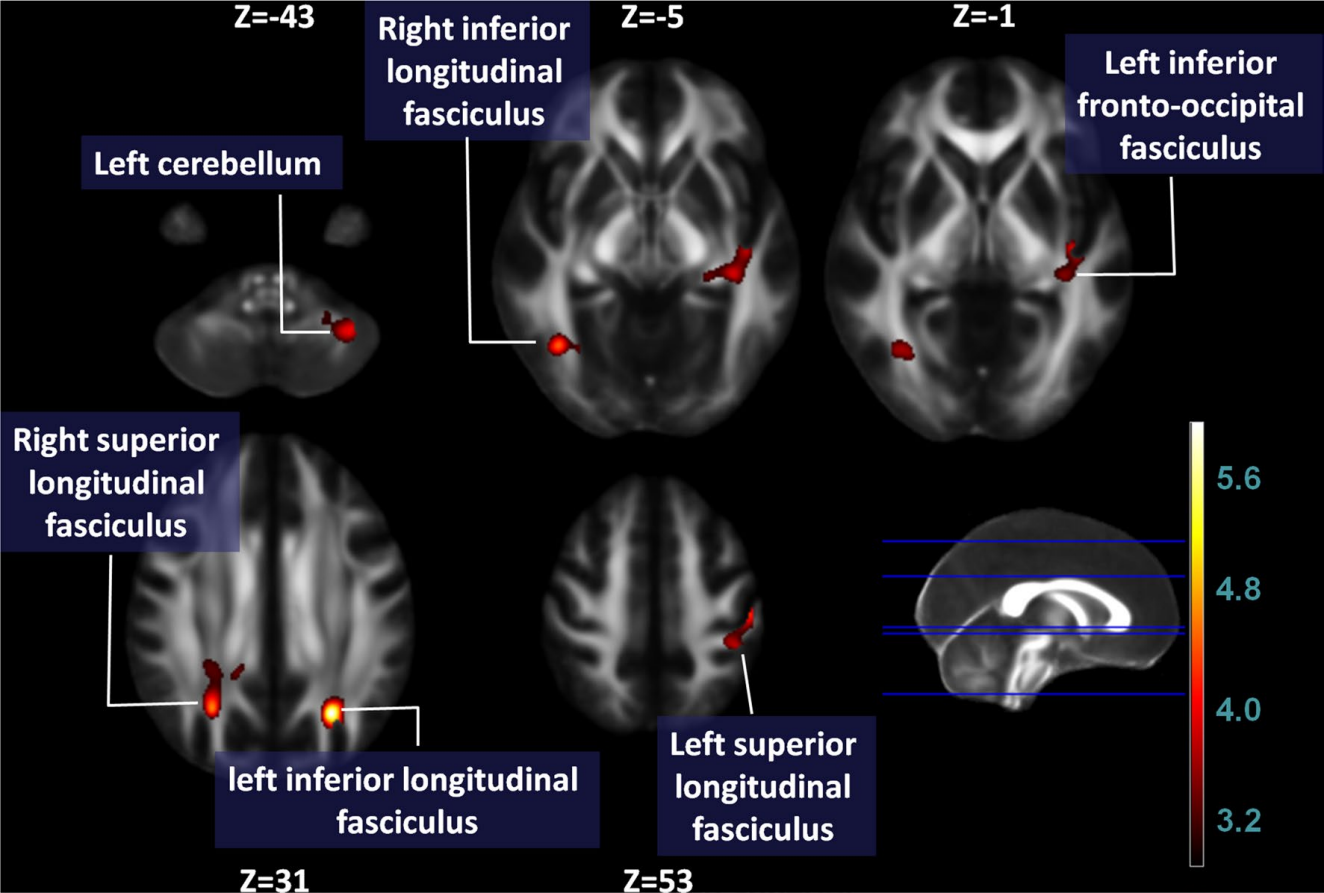
Lower FA values were found in patients with PD (n = 66) versus control subjects (n = 67) in the *left* inferior longitudinal fasciculus (parietal lobe), *right* superior longitudinal fasciculus (parietal lobe), *right* inferior longitudinal fasciculus (occipital lobe), *left* superior longitudinal fasciculus (postcentral gyrus), *left* cerebellum, and *left* inferior fronto-occipital fasciculus (insula)

The location and extent of regions with significant differences in the FA map between the PD and control groups are presented in Table 2. According to the ROI analyses, the patients with PD had lower FA values in the left inferior longitudinal fasciculus (left ILF, parietal lobe), right superior longitudinal fasciculus (right SLF, parietal lobe), right inferior longitudinal fasciculus (right ILF, occipital lobe), left superior longitudinal fasciculus (left SLF, postcentral gyrus), left cerebellum, and left inferior fronto-occipital fasciculus (left IFOF, insula) (Fig. 1).

**Table 2:** Regions showing fractional anisotropy differences among patients with PD and control subjects

MNI Atlas coordinates			Voxel size	White matter tract	Near cortical area	FA, mean ± SD		T_max_	Diffusivity values (PD-NC) (×10^−6^mm^2^/s)		
x	y	z				Controls	PD		MD	AD	RD
*Decreased FA in PD versus controls*											
−25	−63	31	4183	Left inferior longitudinal fasciculus	Left parietal lobe	0.475 ± 0.030	0.445 ± 0.029	6.17	24.83*	1.23	36.64*
26	−60	32	3962	Right superior longitudinal fasciculus	Right parietal lobe	0.467 ± 0.022	0.439 ± 0.026	4.67	24.6*	2.62	35.58*
40	−59	−5	745	Right inferior longitudinal fasciculus	Right occipital lobe	0.430 ± 0.035	0.399 ± 0.038	4.53	15.39	−16.43	31.3*
−41	−21	53	702	Left superior longitudinal fasciculus	Left postcentral gyrus	0.384 ± 0.033	0.361 ± 0.046	4.28	36.41	17.13	46.06
−34	−53	−43	642	Left cerebellum	Left cerebellum	0.270 ± 0.016	0.260 ± 0.017	4.06	21.08*	16.04	23.61*
−38	−17	−1	1423	Left inferior fronto-occipital fasciculus	Left insula	0.429 ± 0.020	0.413 ± 0.023	3.97	26.74*	14.44	32.88*

Location of maximum effect (uncorrected P < 0.001, cluster size >610) was shown in the Montreal Neurological Institute (MNI) space

Group FA mean values in each cluster are presented as mean ± standard deviation

The FA, MD, AD, and RD values in each region of interest were further compared between the two groups by analysis of covariance after controlling for age, sex, and education

*FA* fractional anisotropy, *MD* mean diffusivity, *AD* axial diffusivity, *RD* radial diffusivity, *PD* Parkinson’s disease

* P < 0.05 with the Bonferroni correction, accounting for multiple ROI comparisons

We found that these lower FA values in PD were also associated with differences in other diffusivity indices, including the following: (1) increased MD and increased RD values in the Left ILF, right SLF, left cerebellum, and left IFOF; (2) Increased RD values in the right ILF. No region exhibited significant group differences in terms of AD values.

### Correlations of DTI indices with systemic inflammation and disease severity

Correlation analyses were conducted to evaluate the relationships among the disease severity and systemic inflammation and DTI indices (P < 0.05 for multiple comparisons) (marked as *). The detailed values resulting from those analyses are listed in a Additional file 2: Table S1. In general, aggregated disease severity was associated with poorer DTI indices with decreased FA and increased MD and RD values, and the higher levels of systemic inflammatory markers, including granulocyte apoptosis, lymphocyte apoptosis, LFA-1, and P-selectin, were also associated with poorer DTI indices. Furthermore, a linear regression analysis showed the impacts of various inflammation markers, including granulocyte apoptosis, lymphocyte apoptosis, granulocyte LFA-1, and P-selectin, on the DTI indices (Table 3). The granulocyte LFA-1 contributed to the FA values in the left ILF (β = −0.259), right SIL (β = −0.291), left SLF (β = −0.292), and left IFOF (β = −0.205); the MD values in the right SLF (β = 0.204) and left IFOF (β = 0.151); and the RD values in the left ILF (β = 0.182), right SLF (β = 0.281), right ILF (β = 0.251), and left IFOF (β = 0.163). The granulocyte apoptosis contributed to the FA values in the left ILF (β = −0.218), left cerebellum (β = −0.201), and left IFOF (β = −0.199); the MD values in the left IFOF (β = 0.264); and the RD values in the left ILF (β = 0.178), right SLF (β = 0.201), and left IFOF (β = 0.262). The lymphocyte apoptosis contributed to the FA values in the right ILF (β = −0.184). The total leukocyte apoptosis contributed to the FA values in the right SLF (β = −0.216) and the MD (β = 0.263) and RD values (β = 0.265) in the left cerebellum. The P-selectin contributed to the MD values in the left ILF (β = 0.249) and right SLF (β = 0.188). In general, the granulocyte apoptosis and granulocyte LFA-1 showed the strongest correlation with DTI integrity.

**Table 3:** Linear regression analysis results for correlations between diffusion tensor abnormalities and inflammatory parameters

WM tract	Granu LFA-1		Granu APO2.7 (%)		Lymph APO2.7 (%)		Total APO2.7 (%)		P-selectin	
	Beta (Stand.)	P	Beta (Stand.)	P	Beta (Stand.)	P	Beta (Stand.)	P	Beta (Stand.)	P
*Regression of systemic inflammatory variables*										
FA										
Left inferior longitudinal fasciculus	−0.259	0.002	−0.218	0.008						
Right superior longitudinal fasciculus	−0.291	0.001					−0.216	0.008		
Right inferior longitudinal fasciculus					−0.184	0.040				
Left superior longitudinal fasciculus	−0.292	0.001								
Left cerebellum			−0.201	0.017						
Left inferior fronto-occipital fasciculus	−0.205	0.010	−0.199	0.010						
MD										
Left inferior longitudinal fasciculus									0.249	0.004
Right superior longitudinal fasciculus	0.204	0.018							0.188	0.026
Left cerebellum							0.263	0.002		
Left inferior fronto-occipital fasciculus	0.151	0.039	0.264	0.000						
RD										
Left inferior longitudinal fasciculus	0.182	0.033	0.178	0.034						
Right superior longitudinal fasciculus	0.281	0.001	0.201	0.013						
Right inferior longitudinal fasciculus	0.251	0.005								
Left cerebellum							0.265	0.001		
Left inferior fronto-occipital fasciculus	0.163	0.023	0.262	0.000						

Linear regression between DTI indices and inflammatory parameters was performed after controlling for age and sex as predictors

*APO* apoptosis, *Granu* granulocyte, *Lymph* lymphocyte, *Total* total leukocyte, *LFA*-*1* leukocyte function associated antigen-1, *FA* fractional anisotropy, *MD* mean diffusivity, *RD* radial diffusivity, *WM* white matter

There was no significant correlation between systemic inflammation and disease severity.

## Discussion

In the comparison between the patients with PD and the healthy controls, the patients with PD presented with significantly increased systemic inflammation markers, including increased apoptosis in total leukocytes and its subsets, Mac-1, LFA-1, and serum P-selectin levels. DTI revealed impaired fiber integrity in the patients with PD, indicating increased WM damage. Furthermore, the WM damage was associated with higher disease severity and systemic inflammation. The granulocyte apoptosis and granulocyte LFA-1 showed the strongest correlation with DTI integrity. We may thus say that these two inflammatory markers made greater contributions to the WM damage than the other inflammation variables. The increased systemic inflammation and DTI differences, as well as the associations between them, found in this study help to explain the pathogenesis of altered WM integrity in PD.

Parkinson’s disease is characterized by accumulation of α-synuclein, a kind of misfolded proteins. Recently, more reports point out that inflammation may be the focus for defining disease mechanism [28]. The spread of α-synuclein is not strictly determined by synaptic connectivity, but rather must be dictated by other factors [29]. Alibhai et al. have demonstrated the accumulation of misfolded proteins alone does not define targeting of neurodegeneration, which instead results only when misfolded protein accompanies microglia response [30]. The reactive microglia within the SN are associated with phagocytosing DA neurons and the deposition of α-synuclein in PD [31]. Systemic inflammation contributes to the neurodegeneration in PD via various pathways, and the results here can be attributed to two main pathophysiologies: (1) increased peripheral leukocyte apoptosis and (2) increased expression of leukocyte adhesion molecules.

Higher levels of peripheral leukocyte apoptosis in PD were observed in this study. A review of related studies indicates that leukocyte apoptosis may be either the pathogenesis or the outcome of neuroinflammation in PD. Microglial apoptosis is a sedation process in microglial activation. Through interferon-γ (IFN-γ) signaling, not only is the activation of microglia stimulated [32], but the apoptosis of microglia is also induced [33]. In PD patients, increased IFN-γ in the nigrostriatal DA regions [34] may cross the blood–brain barrier (BBB) into the systemic circulation and cause the apoptosis of peripheral leukocytes.

Mac-1 plays a critical role in reactive microgliosis in PD [4]. Activated Mac-1-positive microglia were previously observed in a mouse model of PD [35], and have been reported to contribute to progressive neurodegeneration, leading to neuronal death [36]. Furthermore, Mac-1 mediates the phagocytosis of degenerated myelin by microglia and macrophages [37]. The recruitment of peripheral Mac-1-positive macrophages through the BBB in CNS demyelination had also been described [38]. A significant increase of peripheral Mac-1 may contribute to increased Mac-1-positive microglia with subsequent neural damage in PD.

LFA-1 contributes to T cell mediated host defense mechanisms. The detailed pathogenesis in PD is not well-known, but the infiltration of increased LFA-1-positive leukocytes in the SN was previously observed in the autopsied brains of 1-methyl-4-phenyl-1,2,3,6-tetrahydropyridine (MPTP)-induced monkeys [5]. A similar result was noticed in the autopsied brains of human PD patients, which presented with increased LFA-1-positive microglia in the SN and putamen, which was, in turn, associated with neuronal degeneration and α-synuclein-positive Lewy neuritis [39]. Increased P-selectin, which plays an essential role in the initial recruitment of leukocytes to the inflammatory site, was also observed in this study, but there have been few related studies discussing its association with PD. The cumulative effects of microglial activation via pathways of IFN-γ, Mac-1, and LFA-1 contribute to DA demyelination and neuritis, which lead, in turn, to the altered WM integrity in DTI. Although IFN-γ, a pro-inflammatory cytokine that contributes microglia activation and causes peripheral leukocyte apoptosis in PD, was not measured in the current study, we could still observe the higher levels of leukocyte apoptosis, Mac-1, and LFA-1 as important factors of WM damage in PD.

Patients with PD show significantly reduced brain FA values, and these changes were found to be localized in various brain regions in our results, which correspond to those of previous studies [14, 40]. The significant WM alterations in PD found in our study can be categorized into three patterns: (1) decreased FA with increased RD and MD, (2) decreased FA with increased RD, and (3) decreased FA only.

The most common pattern of alterations seen in this study was significantly decreased FA with significantly increased RD and MD, along with a non-significant degree of increased AD, in the bilateral parietal lobes, left insula, and left cerebellum, which may reflect myelin and axon loss [41] and may represent chronic WM degeneration [42]. The demyelination of WM fibers disrupts the restricted diffusion in the perpendicular direction, resulting in an increase in RD, and the cellular debris is subsequently cleared by microglia, resulting in an increase in AD [43, 44]. Such a pattern of alterations may be due to the later process of neuroinflammation with WM damage and microgliosis in PD.

Another pattern of DTI index changes consists of significantly decreased FA and significantly increased RD, along with non-significantly increased MD and non-significantly decreased AD, in the right occipital lobe. Such FA reduction along with increased RD has previously been reported to be related to the loss of myelin [45]. Meanwhile, the non-significantly increased MD may be associated with demyelination, while the non-significantly decreased AD may be related to early axonal damage involving axon swelling [43]. Therefore, this pattern of alterations may be due to early axonal damage and demyelination. The only significant DTI index change in the left postcentral gyrus is decreased FA, which may represent a subtle change in WM fiber [46]. The widespread and different WM alterations in the brain may reflect the different stages of the neuroinflammation process in PD.

All of the affected tracts identified in the current study were consistent with those identified in previous reports [14], including the superior and inferior longitudinal fasciculus, the inferior fronto-occipital fasciculus, and the cerebellum. The WM changes in the brain are quite complex. Although we did not observe the WM damage in the anterior part of the inferior fronto-occipital fasciculus in the frontal lobe that has previously been reported in PD with dementia [2], we did observe damage in another part of the inferior fronto-occipital fasciculus in the insular region. This might have been due to the following reasons. First, given the strict conditions of the statistics in DTI indices with Bonferroni correction, only the most severe WM damage would be seen in the results. Second, the PD group in the current study was relatively young and had earlier stages of the disease compared with those in previous WM change studies [47]. Moreover, we did not survey cognitive function in this study, which are better to link the damage in specific brain areas. Relatedly, PD patients without cognitive impairments might not show WM abnormalities compared with healthy controls [48]. And the association between typically recognized “frontal lobe” function and occipital lobe volume suggested a compensatory role of occipital lobe to primary fronto-striatal pathology in PD [49]. Other previous findings not observed in the current study included damage in the internal and external capsule, the corticospinal tract, and the inferior temporal gyrus [14, 15]. In addition to the disease stages, the cognitive function status and movement disorders of the patients involved and the effects of FA statistics [19] may have contributed to these differences between the results of current study and those of previous studies. In conclusion, however, our findings were largely consistent with those of previous investigations of WM damage in PD.

The association between WM damages and systemic inflammation was demonstrated in current and previous studies [50, 51]. The result of linear regression analysis, controlling confounding effects, emphasized the major impacts of systemic inflammation including granulocyte apoptosis, lymphocyte apoptosis, LFA-1, and P-selectin. Although we have discussed the possible interactions of these inflammatory markers with microglial activation and neuroinflammation, the current result cannot identify a definite pathway between systemic inflammation and WM damage. In multiple sclerosis, systemic and neuroinflammation involving T-lymphocyte and microglia have impact on WM damage [52–54]. To the best of our knowledge, the current study is the first to demonstrate a correlation between WM fiber integrity change and leukocyte apoptosis or adhesion molecules in PD. These inflammatory markers may be the possible serum biomarkers of WM damage in PD.

### Limitations

Although the current study has yielded useful findings, its design is not without flaws. First, a control group comprising other neurodegenerative disorders was lacking, and it is possible that the identified inflammatory markers are also associated with other neurodegenerative disorders. Second, some investigators believe that voxel-based statistics are less sensitive than tract-based spatial statistics [14]. However, significant findings, including strong evidence of DTI alterations in PD, were still revealed in this study, in spite of the fact that we used voxel-based statistics to determine those alterations. Third, it should also be noted that the disease severities of the PD patients in this study reflected the relatively early stages of the disease and that nearly twice as many women as men were included in the PD group, whereas men are 1.5 times than women in PD population [55]. Related to above limitations, it is important to emphasize that this was a cross-sectional study, which limits the degree to which its findings can be interpreted as providing a full view of PD, including various complicated details. Future longitudinal studies of this cohort may help, however, to verify the results of this study and clarify the systemic inflammation changes that occur as the disease progresses.

## Conclusions

The patients with PD in this study experienced WM integrity damage in vulnerable regions, and these impairments were associated with increased disease severity and systemic inflammation. The possible interactions among them may represent variant neuronal injuries and their consequent processes in PD.

## Additional file

**Additional file 1:** Supplementary methods of DTI data preprocessing and analysis.

**Additional file 2:** Partial correlation between DTI indices and clinical disease severity.

## Abbreviations

WM: white matter
PD: Parkinson’s disease
DTI: diffusion tensor imaging
FA: fractional anisotropy
AD: axial diffusivity
RD: radial diffusivity
MD: mean diffusivity
DA: dopamine
SN: substantia nigra
Mac-1: macrophage antigen complex-1
LFA-1: lymphocyte function-associated antigen 1
UPDRS: Unified Parkinson’s Disease Rating Scale
Modified H & Y scale: modified Hoehn and Yahr Staging Scale
S & E scale: Schwab and England Activities of Daily Living Scale
PE: phycoerythrin
MFI: mean fluorescence intensity
FMRIB: Functional Magnetic Resonance Imaging of the Brain
ANCOVA: analysis of covariance
ILF: inferior longitudinal fasciculus
SLF: superior longitudinal fasciculus
IFOF: inferior fronto-occipital fasciculus
IFN-γ: interferon-γ
