# Supplementary material for: White matter damage and systemic inflammation in Parkinson’s disease
Source: BMC Neurosci. 2017 Jun 8;18:48. doi: 10.1186/s12868-017-0367-y (PMC5465562; doi:10.1186/s12868-017-0367-y)
Supplement: Supplementary file 1 — Additional file 1: Supplementary methods of DTI data preprocessing and analysis. [file 12868_2017_367_MOESM1_ESM.docx]

**SUPPLEMENTARY METHODS**

***DTI Data Preprocessing***

The magnetic resonance images were preprocessed using the FSL v5.0.4 (Functional Magnetic Resonance Imaging of the Brain (FMRIB) Software Library, Oxford, UK; <http://www.fmrib.ox.au.uk/fsl>), including eddy current correction and brain tissue extraction [[1](#_ENREF_1)]. Each diffusion-weighted image was registered to the nondiffusion-weighted image by an affine registration approach that was supplied in FMRIB’s Linear Image Registration Tool (FLIRT; part of FSL), which was used not only to minimize any image distortion from eddy currents induced by the fast-switching of the gradient coil, but also to reduce any image distortion due to simple head motion. The images were then skull-stripped to remove nonbrain tissue and background noise using the Brain Extraction Tool (BET; part of FSL). A diffusion tensor model was fitted in each voxel using FMRIB's Diffusion Toolbox (FDT, part of FSL) for the calculation of FA values; axial diffusivity (AD) values, λ1; radial diffusivity (RD) values, (λ2+λ3)/2; and mean diffusivity (MD) values, (λ1+λ2+λ3)/3.

The Montreal Neurological Institute (MNI) space served as the standard template space for group comparisons in this study. The FMRIB58_FA standard space image, a high-resolution average of 58 well-aligned good quality FA images in FSL, was used as the target image. Each subject’s FA image was spatially normalized to the target image with a nonlinear registration using FMRIB's Non-Linear Image Registration Tool (FNIRT, part of FSL). The final voxel size of an image was resampled to 1 mm cubic resolution. All normalized FA images were smoothed with an 8-mm Gaussian kernel.

***Analysis of Group Comparison of FA Values***

Statistical analyses were conducted using the SPM8 (Statistical Parametric Mapping; <http://www.fil.ion.ucl.ac.uk/spm/>; University College London, London, UK) software package, which itself used Matlab R2010a (Mathworks, Natick, MA, USA) for voxel-wise group comparisons. Smoothed, normalized FA images were analyzed using SPM8 within the framework of a general linear model, whereas ANCOVA was performed with age, sex, and years of education as covariates to investigate the FA differences between the PD and control groups. The FA threshold of the mean WM was set at 0.2 to successfully exclude voxels, which consisted of gray matter or cerebrospinal fluid in most subjects. The resultant statistical inferences were considered significant under the criteria of cluster level family-wise error (FWE) corrected P value <0.05, with a cluster size of at least 610 voxels, based on the results of the Monte Carlo simulation (3dClusterSim with the following parameters: single voxel P value <0.001, FWHM = 7 mm with GM mask, and 10,000 simulations).

The most probable fiber tracts and anatomic location of each significant cluster were determined using the FSL atlas tool (<http://www.fmrib.ox.ac.uk/fsl/fslwiki/Atlases>).

**References:**

1. Jenkinson, M., et al., *Fsl.* Neuroimage, 2012. **62**(2): p. 782-90.
