# Supplementary material for: White matter damage and systemic inflammation in Parkinson’s disease
Source: BMC Neurosci. 2017 Jun 8;18:48. doi: 10.1186/s12868-017-0367-y (PMC5465562; doi:10.1186/s12868-017-0367-y)
Supplement: Supplementary file 2 — Additional file 2: Partial correlation between DTI indices and clinical disease severity. [file 12868_2017_367_MOESM2_ESM.docx]

**Additional file 1: Table S1.** Correlations between diffusion tensor abnormalities and clinical disease severity

| **Correlation (r) of Clinical Variable** | | | |
| --- | --- | --- | --- |
| WM Tract | UPDRS | H & Y scale | S & E scale |
| **FA** | | | |
| Left Inferior longitudinal fasciculus | -0.414* | -0.373* | 0.424* |
| Right Superior longitudinal fasciculus | -0.392* | -0.351* | 0.319^✝^ |
| Right Inferior longitudinal fasciculus | -0.265^✝^ | -0.240 | 0.238 |
| Left Superior longitudinal fasciculus | -0.208 | -0.246 | 0.250 |
| Left Cerebellum | -0.203 | -0.253^✝^ | 0.201 |
| Left Inferior fronto-occipital fasciculus | -0.203 | -0.203 | 0.182 |
| **MD** | | | |
| Left Inferior longitudinal fasciculus | 0.117 | 0.172 | -0.188 |
| Right Superior longitudinal fasciculus | 0.302^✝^ | 0.336* | -0.279^✝^ |
| Left Cerebellum | 0.301^✝^ | 0.392* | -0.413* |
| Left Inferior fronto-occipital fasciculus | 0.403* | 0.410* | -0.401* |
| **RD** | | | |
| Left Inferior longitudinal fasciculus | 0.279^✝^ | 0.292^✝^ | -0.326^✝^ |
| Right Superior longitudinal fasciculus | 0.374* | 0.384* | -0.329^✝^ |
| Right Inferior longitudinal fasciculus | 0.389* | 0.345* | -0.333^✝^ |
| Left Cerebellum | 0.321^✝^ | 0.409* | -0.428* |
| Left Inferior fronto-occipital fasciculus | 0.407^✝^ | 0.402* | -0.394* |

Correlations between DTI indices and clinical disease severity was performed by partial correlation after controlling for age, sex, and education.

^†^ P < 0.05.

* P < 0.05 with the Bonferroni correction, accounting for multiple region of interest comparisons.
